# Risk Factors for Acute Urticaria in Central California

**DOI:** 10.3390/ijerph18073728

**Published:** 2021-04-02

**Authors:** Rohan Jadhav, Emanuel Alcala, Sarah Sirota, John Capitman

**Affiliations:** Department of Public Health and Central Valley Health Policy Institute, College of Health and Human Services, California State University, Fresno, CA 93740, USA; ealcala@mail.fresnostate.edu (E.A.); shsirota@mail.fresnostate.edu (S.S.); jcapitman@csufresno.edu (J.C.)

**Keywords:** allergy triggers, urticaria, pollution and urticaria, risk factors for urticaria

## Abstract

At least 15–20% of the population in the world suffers from urticaria. Allergy triggers contribute to the development of urticaria. Not much is known about the demographic and environmental risk factors that contribute to the occurrence of acute urticaria. *Methods:* We utilized emergency department data on acute urticaria-related visits managed by the California Office of Statewide Planning and Operations for 201 zip codes located in southern central California (San Joaquin Valley) collected during the years 2016 and 2017. Census data from the same zip codes were considered as a population at risk. Socioeconomic and environmental parameters using CalEnviroScreen (Office of Environmental Health Hazard Assessment, Sacramento, CA, USA) database for the zip codes were evaluated as risk factors. *Results:* The incidence rate of acute urticaria in San Joaquin Valley during 2016–2017 was 1.56/1000 persons (*n* = 14,417 cases). Multivariate Poisson analysis revealed that zip codes with high population density (RR = 2.81), high percentage of farm workers (RR = 1.49), and the composite of those with high and medium percentage of poverty and those with high and medium percentage of non-white residents (RR = 1.59) increased the likelihood of the occurrence of acute urticaria. Conclusion: High population density, farm work, poverty and minority status is associated with a high risk of having acute urticaria.

## 1. Introduction

Urticaria is a common health outcome. Around 15–20% of the population in the world suffers from urticaria [1,2]. Urticaria presents with a sudden appearance of erythematous patches with pruritus [3]. The counts and sizes of patches (wheals) vary [4]. Individual wheal disappear within 24 hours with no treatment [2]. However, new wheals appear on a different part of the body in the case of recurrent episodes of urticaria. Acute spontaneous urticaria episodes lasts for up to six weeks [4]. Acute spontaneous urticaria results from hypersensitivity towards the allergens resulting in the formation of Immunoglobulin E (IgE) antibodies that bind to receptors found on mast cells and basophiles. Upon re-exposure, the allergen is recognized by cell-bound specific IgE, leading to receptor crosslinking. This event leads to the release of many immune mediating enzymes, primarily histamine which causes increased capillary permeability that is presented in the form of swelling on the upper dermal layer of the skin [3]. Common triggers for acute urticaria include viral infections of the upper respiratory track, allergens and psudoallergans, and drugs such as penicillin [1]. Physical urticaria results from a response to physical stimuli. Physical urticaria is further classified into cold, heat, and dermographic and other physical stimuli-specific urticaria. Unlike other physical urticaria where the response to the stimuli is triggered by mast cells deregulation, cold urticaria response can also be triggered by the history of infections, autoimmune diseases and neoplasia [1]. Heat urticaria is rare and is elicited by warm objects or air. Dermographic urticaria results in a swelling of skin along a line that is formed prior to appearance of the swelling (shearing of skin) [5]. Among all physical urticaria subtypes, dermographic form is the most common and is seen mainly among children. Some special forms of urticaria such as cholinergic and contact urticaria are worth consideration, Cholinergic urticaria results from the increase in the body heat usually from the activity such as exercise (exercise induced urticaria). In contact urticaria, the wheals appear at the site of contact of triggers such as chemicals, cosmetics, food products, plants and drugs [1]. 

Studies focusing on urticaria are limited [6]. Women have a higher risk of the onset of urticaria than that among men [7,8,9]. Urticaria is more common in younger adult age groups compared to older age groups [3] However, the effect of a well-known determinant of health, socioeconomic status and factors such as access to care, occupation, and built environment on the likelihood of the occurrence of acute urticaria in particular, is not clear.

Knowledge of specific triggers or causes for the presentation of urticaria is much more developed. Potential triggers include environmental allergens such as pollen, spores originating from mold, dust mites, animal hair [10], food products such as nuts, eggs, fish, seafood, mushrooms, and peas, among others [3], infections particularly among children [11], and physical factors such as cold and hot substance, light, pressure, and water [12].

San Joaquin Valley, the southern part of the Central Valley of California, USA has one of the worst air quality outcomes in the United States [13]. Agricultural infrastructure such as irrigation channels [14], and animal feed and mobile sources contribute to the ozone-induced pollution in the Central Valley [15], in addition to other sources. Studies have shown positive association between air pollution caused by ozone and hospital visits for the treatment of angioedema [16], and the effect of air pollution caused by particulate matter (PM 2.5), ozone and nitrogen oxides on emergency department (ED) visits for the treatment of urticaria [17]. Patients seeking immediate care can utilize ED services without pre-scheduling an appointment with a physician in the United States. Particulates of the smallest aerodynamic sizes (PM 2.5 and lesser) can have detrimental effects on the lungs and heart. The effect of air pollution and the other health outcomes triggered by hypersensitivity to allergens such as asthma particularly in the Central Valley have been addressed [18,19,20]. The prevalence of acute urticaria was more than 50% among those who had allergic asthma or atopic dermatitis in one study [21]. Efforts to understand acute urticaria health outcomes in the Central Valley are limited although the need for such efforts is warranted considering the air pollution and the possibility of a high threshold of environmental allergens originating from agriculture.

The purpose of this study was to identify environmental and sociodemographic risk factors contributing to the visits for the treatment of acute urticaria at emergency departments (ED) of the medical facilities located in the southern Central Valley counties. The study was approved by the institution’s review board for the protection of human subjects. 

## 2. Methods

This study was a retrospective cross-sectional analysis of emergency department visits for acute urticaria. Health data were collected from the Office of Statewide Planning and Development (OSHPD) in California [22]. To maintain a license, hospitals are required to submit emergency department records to OSHPD. De-identified data are made available to the public and researchers within two years of the emergency department visit. Each emergency department record included information on the patient’s gender, age, race/ethnicity, expected source of payment, primary and secondary diagnoses, county, and zip code of residence. For this study, 2016 and 2017 data on emergency department visits were used of individuals residing within the eight San Joaquin Valley (southern area of the Central Valley) counties: Fresno; Kern; Kings; Madera; Merced; San Joaquin; Stanislaus; and Tulare. Zip code-level measures were collected from the US Census (using American Community Survey of 2015–2019) and the California Air Resources Board. 

### 2.1. Outcome Assessment

The incidence rate of urticaria-related emergency department visits was the primary outcome of interest. According to the American Academy of Allergy Asthma and Immunology [23], the diagnosis of urticaria is coded as following:
**ICD-10 Code****Diagnosis**L50.0 Allergic UrticariaL50.1Idiopathic UrticariaL50.2Cold and Heat UrticariaL50.3Dermatographic UrticariaL50.4Vibratory UrticariaL50.5Cholinergic UrticariaL50.6Contact UrticariaL50.8Chronic or Recurrent UrticariaL50.9Unspecified Urticaria

In this study, these codes except L50.4 and L50.8 were used to extract urticaria-related ED events from OSHPD administered data. Allergic urticaria includes acute allergic reaction resulting in urticaria, allergic medicamentosa, and urticaria resulting from the use of a particular food or drug [24]. Idiopathic urticaria is a diagnosis when urticaria erupts and the cause is unknown. Cold and heat urticaria are a result of cold or heat stimuli (physical urticaria), dermatographic urticaria includes autographism, dermatographia, and factitial urticaria. Cholinergic urticaria results from excess body heat, Contact urticaria results from contact with plants or other triggers. Unspecified urticaria is defined as urticaria resulting from exposure to food, drugs, infections, stress and insect bites [24]. The aforementioned urticaria diagnosis codes exclude allergic contact dermatitis, antineurotic edema, giant urticaria, hereditary angioedema, Quincke’s edema, serum urticaria, solar urticaria, urticaria neonatorum, urticaria papulosa, and urticaria pigmentosa [25]. 

In multivariate analysis (Poisson regression), the count of urticaria-related events per zip code was the primary outcome assessed. Zip codes vary in population size; therefore, the population at risk for contracting urticaria varies across zip codes. Population count estimates were used to adjust for the population at risk (offset) within each zip code. This method adjusts the model and allows for urticaria events to be treated as the numerator and the population at risk as the denominator so that regression coefficients can be interpreted as rate ratios. We used patient’s zip code of residence to merge hospital data with the US Census data.

### 2.2. Assessment of Covariates 

We utilized additional data sources to identify the effect of covariates on ED visits for the management of acute urticaria. The percentage of agricultural workers and rurality of a zip code were the two primary covariates of interest. To estimate the percentage of agricultural workers in each zip code, we used 2011–2015 US Census estimates of agriculture, forestry, fishing, and hunting using the American Community Survey. In the 2012–2016 US Census data, population density is estimated by dividing the total population per zip code. Because the average value of population density per zip code was much higher (mean ≥ 19,000 residents per zip code), compared to the average values of other covariates (diesel particulates highest value less than 100 µg/m^3^), we used a natural log of the values of population density. High unevenness in the values of variables can affect the model fit and estimations. The percentage of agricultural workers and population density were treated continuously in analysis. 

For other covariates, additional measures were retrieved from the CalEnvironScreen v1.0 tool, Office of Environmental Health Hazard Assessment, Sacramento, USA which aggregated data to the zip code level from various data sources including the US Census and the California Air Resources Board, among others [26]. Administered by the California Office of Health Hazard Assessment, CalEnviroScreen v1.0 is an environmental and social justice screening tool used in California to allocate cap-in-trade funds to the most disadvantaged communities. A variety of socioeconomic and environmental pollutant measures are used within the tool. The measures used in our study included the percentage of the population living below two times the federal poverty level (percentage of poverty), the percentage of the population that is non-white or Hispanic/Latino (percentage of non-white), the percentage of the population younger than 10 years of age and older than 65, the percentage of the population over the age of 16 that is unemployed and eligible for the labor force (percentage of unemployed), the percentage of the population older than 25 years of age with less than a high school education (percentage of less than high school education), and diesel exhaust particulate matter (Diesel PM). The diesel PM was measured as the spatial distribution of gridded diesel PM from on road and non-road sources for a 2012 summer day in July (kg/day). The California Air Resources Board [27] administers diesel PM emissions data and shares it with the CalEnvironScreen system. We found that percentage of poverty and percentage of non-white had a strong positive multicolinearity effect (Pearson’s correlation value 0.744, *p*-value < 0.05). Therefore, we combined the two variables and used the dichotomous composite in the final multivariate model. This process is referred to as forming a composite to avoid the multicolinearity effect [28]. Multicollinearity can also affect the model estimations. All other variables were treated continuously in the analyses unless stated otherwise and cases with missing data were eliminated from the analysis. 

### 2.3. Statistical Analysis

To fit the discrete nature of the outcome variable, Poisson-based regression was used. Pearson’s *r* and unadjusted Poisson models were conducted to test bivariate associations between each of the predictor variables considered in the analysis and the outcome variable. If the predictor variable was not previously explored in the literature or was not significant, it was not included in the final adjusted model. White’s test of heteroscedasticity demonstrated that an ordinary least squares model was a poor fit for these data (*p* value of <0.001) because of a violation of the assumption of homogeneity of error variance. Therefore, ordinary least squares regression was not used in the final model although it was used in a fully adjusted model to investigate collinearity across predictor variables. Statistical analysis was conducted in the R data analysis system [29].

## 3. Results

There were 14,417 acute urticaria-related emergency department visits from 2016 to 2017 in the San Joaquin Valley composing 0.4% of all ED visits (*n* = 3,237,113). As reflected in Table 1, unspecific urticaria-related ED events had highest attribution towards the distribution of urticaria codes with 73% of all urticaria diagnoses, followed by allergic urticaria-related ED events with 26% of the total. All other urticaria-related events were rare (less than 1%). These results highlight that the dependent variable mainly contained acute spontaneous urticaria. Less than 1% (*n* = 99) of missing data were removed and we successfully merged 14,417 cases with zip codes, and these were used for the analysis. 

As shown in Table 2, a greater percentage of women (56.2%) utilized acute urticaria-related ED services compared to those utilized by men (43.8%). Children younger than five years of age (24.3%) and children aged five to nine (15.1%) had the highest percentage of ED utilization compared to that utilized by any other age group. The mean age of the population was 20.8 years with a standard deviation of 19.3 years. Among racial/ethnic groups, Hispanic/Latino was the most common group for the utilization of ER for acute urticaria treatment followed by white (23.6%) and black/African Americans (5.3%) people. For insurance use type, Medicaid covered the largest percentage of ED visits (69.7%) followed by private insurance (19.5%).

Table 3 illustrates the acute urticaria-related ED visits per 1000 in the population by demographic characteristic. The incidence rate was computed as the total of the number of cases divided by the number of individuals at risk (*n* = estimated 9,226,932), multiplied by 1000. The number of people at risk being the entire population of the selected counties. Overall, the San Joaquin Valley had an incidence rate of 1.56 per 1000 persons from 2016–2017. Women had a higher rate (1.9 per 1000 persons) of acute urticaria-related ED visits compared to those among men (1.5 per 1000 persons). Children less than five years of age had the highest rate of ED visits (5.5 per 1000 persons) followed by those among youth with age less than nineteen years (3.4 per 1000 persons). Black/African American (2.0 per 1000 persons) and Hispanic/Latino (2.2 per 1000 persons) residents had nearly twice the rate of ED visits in comparison to the rate of ED visits among white residents (1.2 per 1000 persons). 

Table 4 illustrates descriptive statistics for the measures included in analyses as well as Pearson’s r for each measure with the outcome of interest. There were 201 zip codes included in the analysis. All measures were treated continuously. In the analysis of bivariate correlations with the rate of urticaria-related ED visits, we found that the percentage of agricultural workers and population density were positively correlated with the rate of urticaria ED visits (Pearson’s r = 0.234, *p* < 0.05 and Pearson’s r = 0.249, *p* < 0.05, respectively). Poverty and non-white concentrations were positively associated with the rate of urticaria ED visits (Pearson’s r = 0.413, *p* < 0.001 and Pearson’s r = 0.393, *p* < 0.001, respectively). Diesel exhaust PM was positively associated with urticaria ED visits (Pearson’s r = 0.166, *p* < 0.01).

In Table 5, the results of a Poisson-based multivariate analysis of the rate of urticaria-related ED visits are shown. Adjusting for covariates in the model, we found that population density was positively associated with ED visits (RR = 2.817, CI = 2.752, 2.883, *p* < 0.05) where one percent increase in population density resulted in 2.81 times the risk of acquiring acute urticaria We found that the percentage of agricultural workers was positively associated with ED visits (RR = 1.490, CI = 1.284, 1.728, *p* < 0.05) where a one percent increase in the percentage of agricultural workers was associated with a 1.49 times increase in the rate of urticaria ED visits. Diesel exhaust PM was significant and positively associated with urticaria-related ED visits (RR = 1.007; CI = 1.005, 1.008, *p* < 0.05) where a one unit increase in diesel PM was associated with a 6% increase in urticaria-related ED visits. The composite of the percentage of those living in low poverty areas and areas with a high percentage of the white population was negatively associated with urticaria-related ED visits (RR = 0.628, CI = 0.600, 0.657, *p* < 0.05). In other words, zip codes (*n* = 61) with low levels of poverty and low levels of non-white individuals have about a 37% lower risk of acute urticaria-related ED visits compared to all other zip codes included in the analysis (*n* = 140).

**Figure 1:**
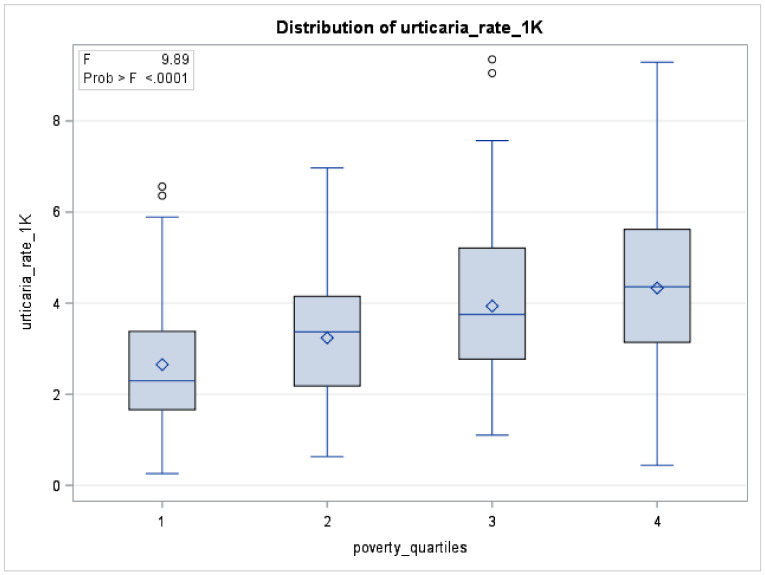
Distribution of acute urticaria rate per 1000 residents by poverty quartiles for selected zip codes (*n* = 201), San Joaquin Valley, 2016–2017. Boxplots for percentage of poverty quartiles 1–4 are shown (F < 0.0001).

As shown in Figure 1, acute urticaria rates were higher in zip codes with higher levels of poverty. 

**Figure 2:**
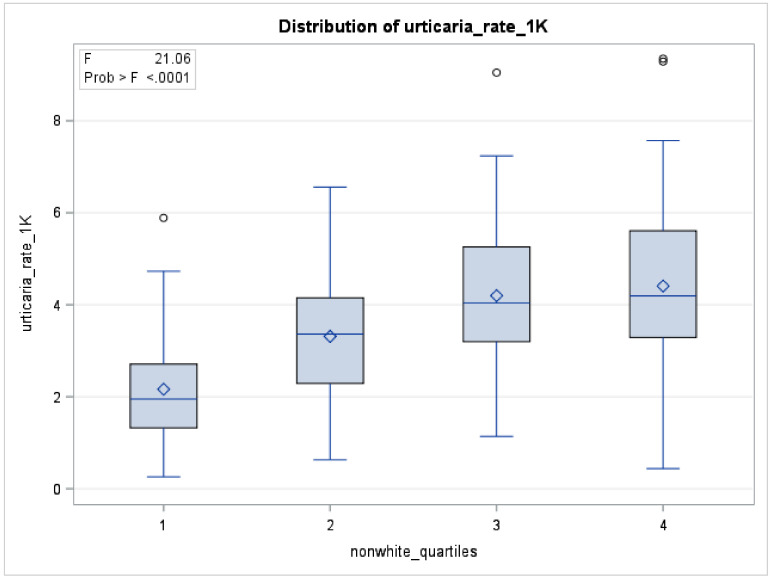
Distribution of acute urticaria rate per 1000 residents by percent of non-white residents’ quartiles for selected zip-codes (*n* = 201), San Joaquin Valley, 2016–2017. Boxplots for percentage of non-white quartiles 1–4 are shown (F < 0.0001).

As shown in Figure 2, acute urticaria rates were higher in zip codes with higher percentage of non-whites. 

## 4. Discussion

### 4.1. Prevalence of Urticaria 

San Joaquin valley presents a unique environment. The valley has a semiarid climate with very hot and dry summers and mild winters. As America’s breadbasket, agriculture and related industries are the most common among the industries in the valley. Exposures resulting from agricultural operations such as dust and pesticides add to the pollution in addition to the pollutants from other natural and manmade sources including frequent bushfires in California. Allergens from air pollution have contributed to an increase in asthma outcome in the Central Valley [18,20]. However, other outcomes associated with allergens in air pollution in the Central Valley have not been previously studied. Therefore, we attempted to understand the status of one of the non-asthma allergy outcomes such as acute urticaria in the Central Valley. In our study, we found that the incidence rate of ED visits for acute urticaria was 1.56 per 1000 persons during 2016–2017. Acute spontaneous urticaria was attributed to 0.4% of all emergency visits in the selected central California counties during 2016–2017. These findings are comparable to other studies. In an Italian study, the authors reported that acute urticaria represented 1.01% of all ER visits [30]. In a large cross-sectional study, the prevalence of acute urticaria among children participants was reported as 13.9% [31]. The reason for such discrepancy between ours and this study could have emerged from our analysis method where we used the population at risk at the zip code level from the selected counties. 

### 4.2. Risk Factors for Acute Urticaria

#### 4.2.1. Working in Agriculture

In our study, we found that working in agriculture increased the risk of acute urticaria. Multiple studies have reported contrary evidence. In a Finnish study based on an analysis of national level physician-reported data, the authors found that agricultural workers were the most common occupational group with contact urticaria and that cow dander was the most commonly found cause of contact urticaria [7]. In one study, the authors reported that among vocational agricultural students, the incidence of dermatological allergic outcomes was 6% and that 3/4th of the cases had urticaria [32]. In a review of studies focused on allergic conditions in agriculture occupation, authors found that urticaria was among the most commonly diagnosed health problem among farmers [33], and that ever-changing agricultural practices pose new sets of allergens. Most agricultural workers lived in suburban settings. It’s also possible that the environment (air pollution etc.) in these settings may have brought agricultural workers close to infections and allergies. Future studies should explore more deeply into agricultural worker living conditions and the risk of allergic diseases including acute urticaria. Also the risk of acute urticaria should also be characterized by the type of farm (dairy, machinery, mixed), commodities (soybean and corn, fruits and vegetables, nuts etc.), farm size (large, small, medium), region (Midwest, South, West, South), and other appropriate distinctions. 

#### 4.2.2. Urban Indicator 

In our study, we found that living in an urban setting increased the risk of having acute urticaria. A Polish survey reported no effect of urban lifestyle on the first episode of urticaria [2]. Our result is in line with a Brazilian study [9]. Authors in another study also reported higher incidences of allergic diseases among those living in urban area in comparison with rural residents [34]. Urban and rural environments can have mixed set of exposures and allergens. In some cases, city and town residents are exposed to allergens from pollutants originating from factories and vehicles a lot more frequently compared the exposure received by their rural counterparts. However, in other cases air pollution could have been resulted from natural as well as anthropogenic sources in both settings. For example, a study demonstrated that in an urban setting in the Central Valley of California, air pollution was driven by highway and street traffic as well as nearby agricultural sources [35]. The agricultural sources contributing to PM 2.5 and PM 10 production may also include manmade sources such as pesticides. While in urban settings, natural sources of pollution could be significant as well. In a study from Tokyo, Japan, the authors showed that PM 2.5 modified the association between exposure to pollen and pollinosis (hay fever) [36]. Like the results reported in this study, it is possible that diesel exhaust PM 2.5 could be attracting more pollens and thereby resulting in high risk of acute urticaria among the urban residents in our study. 

#### 4.2.3. Percentage of Poverty 

We showed that the individuals living in zip codes that have high and medium percentage of poverty level had a higher risk of urticaria-related ED visits compared to the individuals living in zip codes that have a low percentage of poverty level. As shown in Figure 1, the rate of ED visits for urticaria increased with the increase in the poverty level. A study from China reported similar findings. They reported that a low parental income of 18-year-old study participants was associated with high risk of chronic spontaneous urticaria [37]. However, a nationwide retrospective cohort study from Taiwan reported contrary evidence. According to this study, children with high family income had higher risk of chronic urticaria compared to children with lower family income [31]. Poverty is a well-recognized determinant of health. Poverty described in our study is a zip code level observation which precisely helps identify the specific neighborhoods with high and medium percent of poverty. When the neighborhoods have high poverty level, residents often face numerous challenges although they themselves may not be poor [38]. Because of the spatial nature of this result, it helps depict the ground level situation of an uneven distribution of percent of poverty across the regions that translates into high urticaria risk. In other words, living in such neighborhoods could bring them closer to infections and allergens resulting in high incidence of acute and chronic allergic diseases including urticaria.

#### 4.2.4. Percentage of Non-White Race

In our study, individuals living in zip codes with a high and medium percentage of non-white neighborhoods had a higher risk of urticaria-related ED visits compared to the individuals living in zip codes with low percentage of non-white neighborhoods. As shown in Figure 2, the risk of acute urticaria increased with the increase in the percentage of non-white residents from the selected zip codes. To our knowledge, no other study addressed race as a risk factor for acute urticaria. Chronic spontaneous urticaria has been found to have a higher prevalence among non-whites [36]. It may be possible that non-whites live in neighborhoods that consists of high exposure to allergens and pseudoallergens. However, because of the difference in the pathogenesis for acute and chronic urticaria, alternative explanation could be suggested for the high prevalence of acute urticaria. We attempted an interaction between diesel exhaust and the percentage of non-whites for urticaria rates per 1000 residents. The interaction was not statistically significant. Further studies should identify the set of allergens among non-whites that lead to high risk of acute and chronic urticaria. Challenges contributing to the risk of occurrence of acute urticaria among non-whites such as difficulty of access to healthcare, income disparity, and environmental justice issues, among others should also be explored by further research.

#### 4.2.5. Percentage Less Than 18 Years of Age 

In our study, we found that being less than 18 years of age increased the risk of developing acute urticaria. In a Polish population survey, the authors reported that the mean age for the first episode of acute urticaria was 15 years [2]. An Australian study based on the analysis of large datasets on hospital admissions during 1993–1994 and 2004–2005 reported a high risk of food-related anaphylaxis among children less than five years old [8]. In our study, the proportion of acute urticaria was highest among children less than five years old followed by children aged 5 to 10 and children aged from 10 to 14. Urticaria is very common reason for pediatric visits [37]. Acute urticaria among adults requires antihistamine treatment [38] while acute urticaria among children is usually caused by infections [39]. Of all urticaria-related ED counts in our study, 48.3% were attributed to children aged 14 years or less. Therefore, infections may have contributed substantially to acute urticaria-related visits in our study. However, this possibility can be verified by future studies.

#### 4.2.6. Gender

In our study, we found that the rate ratio was nearly 3:4 for male to female. Others reported similar results. Salvarates et al. [9] reported 1:3 ratio for chronic urticaria angioedema. A Finnish study reported that occupational contact urticaria was much more common (70% of total cases) among women compared to the cases among men [7]. A Polish study showed that the incidences of acute urticaria were more likely among women compared to those among men [2]. An Australian study showed 43% and 45% of the total number of cases admitted in the hospital for angioedema and urticaria during 1993–1994 and 2004–2005 were men [8]. The same study showed the hospital admissions for angioedema and urticaria were more common for boys than girls. 

#### 4.2.7. Diesel Particulate Matter 

In our study, we found a positive association between diesel particulate matter and increased incidence of ED visits for acute urticaria. According to the International Agency for Research on Cancer, diesel exhaust is a probable carcinogen (group 2A) [40]. Particulates originating from the combustion of diesel fuel have been shown to produce immediate immune responses among patients with asthma [41,42]. In a Canadian study, the researchers analyzed hospital data consisting of 2905 ED visits for urticaria using case crossover design. They found a positive association between the ambient quality health index consisting of measurements of PM 2.5, ozone and nitrogen oxides and urticaria-related ED visits [17]. Researchers from Lebanon found that PM 2.5 and PM 10 pollutants were associated with the development of urticaria among children [43]. Not much information on the role of air pollution and traffic particulates on developing the triggers for urticaria is available. The air pollutant PM penetrates the skin through diffusion [44] which leads to the production of proinflammatory cytokines. The PM also causes oxidative stress to dermal cells and the release of radioactive oxygen species (ROS). The ROS play a vital role in the development of acute urticarial symptoms [45]. 

### 4.3. Strengths

To our knowledge, no other study has addressed the urticaria situation in the Central Valley of California; the unique environment that might be contributing to the triggers for different allergic conditions. Another major strength of this study was the application of combined ED visit data and the census data using the American Community Survey resulting in estimates that are more generalizable compared to the situation where analysis restricted to ED visit data only. The possibility of self-reporting bias was also eliminated because of the use of ED data.

### 4.4. Limitations 

The study had some limitations. The outcome variable was the ED visits count for acute urticaria and the visits could have been first time or recurrent for the individual patient. We did not account for the specific allergic triggers that could exacerbate urticaria episodes other than diesel particulates. Further efforts should focus on explaining the cause-and-effect relationship for the triggers that could be unique to the Central Valley. As opposed to a two-year data in our study, a five-year or more data can present opportunities to understand the trends of acute urticaria. 

## 5. Conclusions

In this population-based study based in the Central Valley of California, we found that high and medium percentage of poverty and high and medium percentage of non-white population, greater percentage of agricultural workers, young age (less than 18), and higher concentration of diesel exhaust were associated with a high risk of acute urticaria. In particular, the magnitude of the risk of acute urticaria was highest among zip codes with high population density, high percentage of agricultural workers and those containing high and medium percentage of individuals living in poverty and had high and medium percentage of racial minorities. Furthermore, it is possible that most agricultural workers lived in zip codes with high and medium percentage of poverty and the greater to medium percentage of racial minorities. With the unique situation of farming practices (production of diverse commodities) and workforce (substantial amount of migrant and minority workers) in California compared to the other regions in the United States, it is important to understand the needs of farm workers as they may have these environmental and social challenges that could put them at risk for acute urticaria. Further studies should explore more about agricultural worker living conditions, access to care and economic situation, and should also emphasize designing intervention to reduce acute urticaria outcomes targeted for other communities such as those living in urban and suburban neighborhoods with high social and economic inequalities.

## Figures and Tables

**Table 1:** Urticaria-related emergency department visits by urticaria diagnosis codes San Joaquin Valley, 2016–2017.

Urticaria Diagnosis Code	Frequency (*n*)	Percentage (%)
L50.0 (Allergic Urticaria)	3717	25.60
L50.1 (Idiopathic Urticaria)	101	0.70
L50.2 (Cold and Heat Urticaria)	4	0.02
L50.3 (Dermatographic Urticaria)	15	0.10
L50.5 (Cholinergic Urticaria)	2	0.01
L50.6 (Contact Urticaria)	11	0.07
L50.9 (Unspecified Urticaria)	10,567	72.80
Missing Data	99	0.70
Total	14,516	100.00

**Table 2:** Frequency and percentage by demographic characteristics of the population with an acute urticaria-related emergency department visit, San Joaquin Valley, 2016–2017.

Demographic Characteristic	*n*	%
Gender		
Women	8100	56.2
Men	6317	43.8
Age		
Younger than 5 years of age	3505	24.3
age 5 to 9	2179	15.1
age 10 to 14	1285	8.9
age 15 to 19	1239	8.6
age 20 to 24	1200	8.3
age 25 to 29	1043	7.2
age 30 to 34	801	5.6
age 35 to 44	1197	8.3
age 45 to 54	865	6.0
age 55 to 64	609	4.2
age 65 to 74	317	2.2
age 75 to 84	141	1.0
age 85+	36	0.2
Race/Ethnicity		
White	3397	23.6
Black/African American	764	5.3
Hispanic/Latino	9041	62.7
Asian/Pacific Islander	711	4.9
Other Race/Ethnicity	504	3.5
Insurance		
Medicaid	10,053	69.7
Medicare	680	4.7
Other Payer	174	4.7
Private	2837	19.5
Self-Pay	673	4.7

**Table 3:** Rate of acute urticaria-related emergency department visits by demographic characteristic, San Joaquin Valley, 2016–2017.

Demographic Characteristic	Rate Per 1000
Gender	
Women	1.9
Men	1.5
Age in Years	
Younger than 5 years of age	5.5
Persons younger than 19	3.4
Persons 65 years and over	0.5
Race/Ethnicity	
White	1.2
Black/African American	2.0
Hispanic/Latino	2.2
Asian/Pacific Islander	1.1
Other Race/Ethnicity	0.4

**Table 4:** Descriptive statistics of zip code-level (*n* = 201) measures for counts of acute urticaria, San Joaquin Valley, 2016–2017.

Measure	Min	Max	Mean	SD	Pearson’s *r* with Rate of Urticaria
% agricultural worker	0	75	17.92	18.10	0.234 **
Population density	0	1	0.25	0.43	0.249 **
Diesel exhaust PM	0.71	68.60	8.69	12.16	0.166 *
% less than 18 years of age	11.69	42.31	26.90	3.30	0.102
% non-white	12.38	98.48	60.47	24.76	0.413 ***
% poverty	0	88.1	47.96	19.35	0.393 ***
Count of acute urticaria	1	432	73.57	84.54	-
ln (population at risk)	6.03	11.32	9.21	1.36	-

*** *p* < 0.001, ** *p* < 0.01, * *p* < 0.05. Pearson’s *r* was used to test bivariate correlations.

**Table 5:** Results from Poisson-based multivariate analysis on the counts of acute urticaria for the selected zip codes (*n* = 201), San Joaquin Valley, 2016–2017.

Parameter	*beta*	95% CI	RR	95% CI
Intercept	−6.394 ***	(−6.710, −6.078)	0.002	(0.001, 0.002)
Diesel particulate matter	0.006 ***	(0.005, 0.007)	1.006	(1.005, 1.008)
Population density	1.036 ***	(1.012, 1.059)	2.817	(2.752, 2.883)
% agricultural worker	0.399 ***	(0.250, 0.547)	1.490	(1.284, 1.728)
% less than 18 years of age	0.015 ***	(0.008, 0.022)	1.015	(1.008, 1.022)
Composite of lower % of non-white and poverty (dichotomous)	−0.466 ***	(−0.511, −0.420)	0.628	(0.600, 0.657)

*** *p* < 0.05.

## Data Availability

Emergency Department data for 2016–2017 can be requested from California Office of Statewide Planning and Development, Sacramento, California, United States using the following link: https://oshpd.ca.gov/data-and-reports/research-data-request-information/. De-identified datasets are available to researchers upon request. For covariates, publicly available American Community Survey data for the same zip codes were used using the link: https://www.census.gov/acs/www/data/data-tables-and-tools/data-profiles/.
